# Effects of textured food masticatory performance in older people with different dental conditions

**DOI:** 10.1186/s12877-022-03064-w

**Published:** 2022-05-02

**Authors:** Young-Sook Park, Han-Pyo Hong, Soo-rack Ryu, Suyong Lee, Weon-Sun Shin

**Affiliations:** 1 Department of Food & Nutrition, College of Human Ecology, Hanyang University, 222 Wangsimni-ro, Seongdong-gu, Seoul, 133-791 South Korea; 2 Biostatistical Consulting and Research Lab, Medical Research Collaborating Center, Hanyang University, Seoul, South Korea; 3 Department of Food Science and Biotechnology, Sejong University, Seoul, South Korea

**Keywords:** Mastication, Older people, Dental status, Tongue pressure, Texture-modified food

## Abstract

**Background:**

Physiological deterioration (aging, poor dental status, and reduced tongue pressure) makes chewing difficult. This study aimed to investigate the chewing patterns of older people with or without dentures, evaluate the textural and masticatory properties of texture-modified radish *Kimchi*, and investigate the correlation between dental status and tongue pressure. Additionally, differences in the subjective–objective concordance of texture-modified *Kimchi* were investigated using the preference test.

**Methods:**

This study included 32 Korean women aged between 65 and 85 years. Masticatory behavior was recorded by electromyography, and tongue pressure was measured using the Iowa Oral Performance Instrument. A preference test, with hardness as the relevant textural property, determined the participants’ preferences among the test samples (food with a homogeneous structure—radish *Kimchi*). To assess preference differences, a questionnaire suitable for older people was designed. The preference for cooked radish *Kimchi* with various blanching times based on overall acceptability and self-reporting of preference was investigated to develop elderly-friendly food. The subjective scores indicated whether the sample (radish *Kimchi*) was hard or soft based on the chewing ability of the participants. Dental status, muscle activities, and tongue pressure were considered for the food design with optimized texture. The relationship between subject score and mastication properties were examined using multiple regression analysis.

**Results:**

The number of chews and chewing time increased with hardness, significantly activating the masseter and temporalis muscles. The evaluation of masseter muscle activity, particularly for level-6 radish *Kimchi*, showed that older people with complete dentures chewed less actively than those with natural teeth (*p* < 0.05). The older people with natural teeth (18.94 ± 10.27 kPa) exhibited higher tongue pressure than those with complete dentures (10.81 ± 62.93 kPa), and the difference was statistically significant (*p* < 0.01). Older people preferred food with familiar tastes and textures. An association was found between the subjective hardness score and the objective hardness level. The perceived hardness intensity was linked to the chewing ability of the participant. Denture wearers exhibited a lower chewing ability, and at level 6, they perceived greater hardness of food than those with natural teeth.

**Conclusions:**

Developing food with a modified texture can bridge the gap between physiological and psychological aspects of food texture; texture-modified radish *Kimchi*, with limited blanching time, may be favorable for older people.

## Background

The primary function of mastication is to form a bolus that can be swallowed safely [1]. Mastication involves not only the breaking down of solid food but also changing its consistency to enable easy swallowing [2]. Chewing or breakdown of food by the teeth to a condition suitable for further transport to the esophagus is important [3]. Therefore, after the food enters the mouth, it is processed (mastication) and then swallowed. Chewing is the first step of the digestion process and prepares food for swallowing and processing in the digestive system [4]. Food oral processing is also essential for sensory perception [5]. Oral health is important for maintaining good nutritional status in older poeple [6]; aching and mobile teeth, as well as poor-quality prostheses, may affect masticatory behavior, leading to decreased digestive function [7]. Moreover, masticatory efficiency decreases in people with missing teeth [6]. In fact, many elders experience decreased digestive function and difficulties in swallowing due to missing teeth caused by poor oral health. Additionally, masticatory dysfunction leads to swallowing difficulties and undernutrition [8].

The masticatory process can be analyzed by electromyography (EMG), which measures parameters such as the time of chewing activity, the number of chewing cycles, and the masticatory muscle activity. EMG techniques have been used for 30 years, mainly to analyze the link of mastication mechanics to sensory properties [9]. Notably, studies [4–6, 10, 11] have identified the natural chewing pattern of individuals using EMG of the masticatory muscles. Similar to most EMG studies, many of the cited studies have the caveat of low sample sizes. However, this is nearly unavoidable in EMG research owing to the time consuming and costly nature of data collection. Therefore, the interpretation of the results of these studies should be analyzed within the context of their individual limitations.

Recently, clinical studies have applied various techniques to measure tongue-palate compression using the Iowa Oral Performance Instrument (IOPI), which has been used to investigate the influence of aging and sex on tongue performance and tongue pressure generation during swallowing [12, 13]. Accumulating evidence [6, 8, 12] has emphasized that aging is associated with physiologically weakened muscles, decreased teeth number and movement coordination, and difficulties in eating. For older people, soft texture of food is a key factor for easy chewing and swallowing. Older people naturally adjust to hard-textured food by using methods which allow them to continue enjoying the foods, e.g., dunking hard biscuits in tea to soften them prior to eating [14]. Nevertheless, they tend to be unwilling to give up solid food, even when it is not adequately soft to facilitate the chewing process. For instance, traditional radish *Kimchi* (*Kkakdugi*) is popular among older-Koreans, who have eaten it as a prominent side dish throughout their life. Generally, older people have well-established food preferences and are more likely to maintain traditional eating habits [15]. However, despite their preference, dental conditions often cause difficulty in chewing and swallowing solid food, such as radish [16]. Hence, it is necessary to provide food with modified texture for older-individuals with decreased eating capability. Notably, blanching can be used to soften solid food, making mastication much easier [17]. Previous studies focusing on food texture [16, 18] have identified the key mechanisms for the perception of hardness and texture of food in relation to their intrinsic properties and breakdown in the mouth. Some of the earlier studies [18–20] have focused on several food items with a macroscopically heterogeneous structure (e.g., cream-filled biscuits and yogurt with fruit pieces). In contrast, other studies [21–23] on food matrices have focused on foods which are homogeneous structured food (e.g., jelly confectionery products, biscuits, roast pork). In our study, we have used radish, which has a homogeneous matrix with a characteristic hard exterior and interior. Studies [23, 24] have extensively investigated traditional Korean food such as radish *Kimchi*. However, to the best of our knowledge, no study has examined the relationship between homogeneous hard textural radish and dental statuses.

We hypothesized that partially texture-modified radish *Kimchi,* with no change in taste and flavor, can be useful for older, and those with masticatory difficulties would prefer partially texture-modified radish *Kimchi*. Accordingly, the present study aimed to evaluate the textural and masticatory properties of texture-modified radish *Kimchi* and investigate the correlation between the dental status and tongue pressure. Additionally, differences in the subjective–objective concordance of texture-modified *Kimchi* were investigated using the preference test. We expect that older people prefer familiar tastes and textures in their daily diet and, therefore, texture-modified food is needed to match their chewing capability.

## Methods

### Participants

A total of 32 Korean women between 65 and 85 years of age at the Seoul Welfare Center were included in this study. Participant recruitment was conducted between December 2017 and February 2018. The recruitment involved healthy older volunteers. ​Of these older people, those who were capable of independent activities of daily living and following instructions were included in this study.

All the procedures were conducted at the School of Hanyang University in Korea. Participants were informed about the aims, methods, and safety issues of the test, and all of them provided their written consent before participation in this study. This study was approved by the Ethics Committee of Hanyang University (HYI-17–198-4).

Participants visited Hanyang University once, and demographic questions concerning their sex and age were obtained. Participants provided self-assessed responses concerning their ability of independent living and experience of any dental problems; these responses contributed to the selection process. Subsequently, the older participants were classified into two groups according to their dental status: those with natural dentition (not partial denture wearers, *n* = 24) and those wearing a full denture (*n* = 8).

None of them had any experience with EMG or IOPI experimentations; thus, detailed instructions were provided. Participants had to attend a single 1-h session, and each experiment lasted 1 h.

### Test food

The test food was provided by Pulmuone (Seoul, Korea). The radishes were washed with water to remove impurities and then cut into uniform cube-shaped pieces (1.5 × 1.5 × 1.5 cm) to avoid cooking-related differences; the thick peel was not used. Subsequently, the cubes were blanched in boiling water for different durations (0, 3, 4.5, 7, 9, or 15 min). Next, the cubes were mixed with salt and other ingredients. The sample *Kimchi* consisted of 90% radish, 3% onion, 2% red chili pepper powder, 1.5% minced garlic, 0.6% salt, 0.5% anchovy sauce, 1.0% salted shrimp, 0.3% minced ginger, and 1.1% white sugar.

The radish *Kimchi* (*Kkakdugi*) was made with raw white radishes. Subsequently, the samples were packed into a jar after thorough mixing to obtain a homogeneous sample.

For statistical analysis, a total of six kinds of test food samples were prepared, including hard radish *Kimchi* as the control and various types of texture-modified cooked radish *Kimchi*, which were softened by blanching.

### Instrumental texture measurement

Puncture tests were performed using a texture analyzer (TAXT plus, Stable Micro Systems, Surrey, UK) to assess the textural characteristics. In this test, the diced radish cubes (1.5 × 1.5 × 1.5 cm) were tested in the texture analyzer with a 100-N load cell to assess their hardness (in N). For the puncture test, a probe rod (5 mm in diameter) was pressed into each sample at a speed of 100 mm/min, and the peak force was assessed [23]. All texture analyses were performed at room temperature with two replicates.

### EMG measurements

EMG was performed to assess masticatory properties, and the participants were asked to sit upright in a chair to eat the test food. After carefully cleaning the skin surface, pairs of surface electrodes (T246H, Bioprotech, Daejeon, Korea) were attached to the skin on the left and right masseter muscles and temporalis muscles, and the electrodes of a 4-channel EMG device (LXM 5308, Laxtha, Daejeon, Korea) were also attached over these muscles. Subsequently, the participants were asked to chew and comfortably swallow food as usual. They were required to have a minimum of two chewing cycles and be free of interruptions, such as talking or spitting out the food. For testing, refrigerated samples (4 °C) were dispensed into a plastic dish with six wells and served after 5 min at room temperature. Each participant was presented with food samples varying in hardness—softest (blanched for 15 min) to hardest (no blanching)—in a plastic dish. The six samples of different hardness levels were presented to participants one at a time in the six-well dishes. Subsequently, the individuals chewed and swallowed all *Kimchi* samples (levels 6 to 1); they took a brief rest to reduce fatigue between the duplicate measurements. By placing sensors on their faces, muscle activity during jaw opening and closing were measured; thus, chewing patterns could be captured during food consumption.

The EMG recordings were performed from the time of ingestion of food until deglutition of the bolus.

The data were classified based on each test food and were analyzed with scripts developed using TeleScan version 2.99 (Laxtha, Daejeon, Korea). For statistical analysis, the total number of chews, the sum of chewing cycles to finish the test food, and the chewing time were monitored [16]. When participants finished eating and orally processing the stimulus, they raised their hands to indicate the end point of eating.

In this way, the total eating time was self-reported and included multiple swallows and the oral clearance time needed to remove residues or oral coatings from the tongue surface.

The chewing time was defined as the duration of chewing of a test food from ingestion until completion of oral clearance [13].

The electromyographic potentials of the left and right masseter and temporalis muscles were recorded, and the mean EMG amplitude was calculated. The EMG voltages across each electrode were averaged.

Peak-to-peak amplitudes, durations, and muscle activities (time-integrated EMG voltages) were calculated for each muscle according to its actions [11].

### IOPI tongue pressure measurements

The participants’ tongue pressure and strength were measured using the IOPI (TPS 100, Cybermedic, Gwangju, Korea). Especially, tongue-palate compression based on IOPI values was assessed.

Participants were asked to sit in the most relaxed posture, with their head upright and eyes focused on a target at the horizontal level. They were required to press an air balloon probe between the tongue and hard palate as hard as they could by closing their mouth to reach peak values. Individuals were then asked to press the balloon probe using the maximum effort for several seconds until the sound signal stopped. Brief rests between the triplicate measurements were offered to reduce fatigue [25].

For statistical analysis, the generated tongue pressure was recorded in kilopascal (kPa), and tongue-palate compression was determined as a function of the maximum isometric tongue pressure (MITP) generation capacity using the IOPI [26]. Moreover, the mean peak values for the three records were defined as the average pressure level (APL).

### Preference test and hardness sensitivity

To assess differences in the preference of the food samples, a questionnaire suitable for older was designed. The preference for cooked radish *Kimchi* with various blanching times was investigated to develop elderly-friendly food. For the preference test, each participant self-reported the choices of their favorite test samples based on overall acceptability.

Then, the hardness sensitivity of the participants was evaluated by asking them to assess food as “hard” or “soft.” Participants also rated the intensity of “hard” and “soft” using a 9-point category scale (1 = extremely soft, 2 = very soft, 3 = soft, 4 = slightly soft, 5 = moderately, 6 = slightly hard, 7 = hard, 8 = very hard, and 9 = extremely hard).

### Statistical analysis

SPSS software (IBM SPSS Statistics for Windows, version 20.0; IBM Corp., Armonk, NY, USA) was used for the statistical analyses. A *p*-value of < 0.05 was considered statistically significant.

One-way analysis of variance (ANOVA) was used to compare EMG activity based on changes in radish *Kimchi* hardness. Data were analyzed by one-way ANOVA, followed by Bonferroni correction. One-way ANOVA was applied for the analysis of each test food.

Comparisons between the two groups (older-individuals with natural teeth and those with full dentures) were performed using Bonferroni correction. In addition, masseter muscle activities and tongue pressure were compared between the two groups (older individuals with natural teeth and those with full dentures) using t-tests.

Pearson correlations were conducted to explore inter-relationships for masticatory parameters. Thereafter, multiple regression analysis was performed to assess the influences of subject score on masticatory performance.

## Results

### Hardness of radish *Kimchi*

Blanching in boiling water for 0, 3, 4.5, 7, 9, and 15 min was used to modify the texture of the radish *Kimchi*. The hardness of the blanched radish *Kimchi* was measured at ambient temperature. The blanched radish *Kimchi* was categorized into six types based on hardness level. Naturally, a longer blanching time was associated with softer textures of the radish *Kimchi.* Subsequently, to evaluate the chewing performance of the older people for the blanched radish *Kimchi*, the masticatory characteristics of the participants were monitored by EMG, whose signals can be collected during mastication.

### Number of chews and chewing time according to hardness changes in radish *Kimchi*

**Fig. 1:**
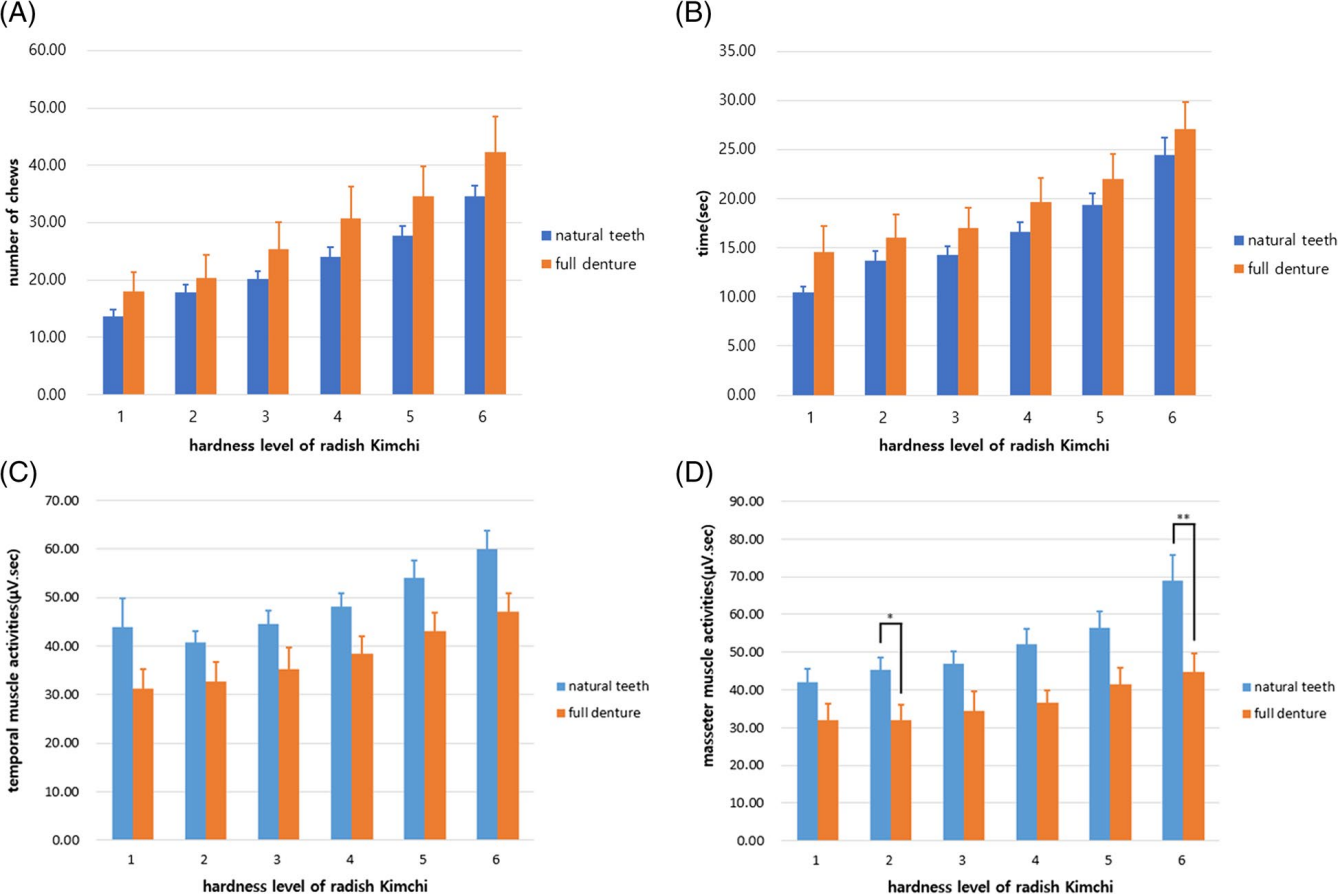
Number of chews (A) and chewing time (B) according to the test food hardness. Temporal muscle activity according to the test food hardness (C). Masseter muscle activities (μV.s) in natural teeth and full-denture participants according to the hardness of the test food (D) *p < 0.05, **p < 0.01

As the hardness of the radish *Kimchi* increased, the number of chews increased significantly among older individuals with natural teeth or a full denture (Fig. 1A). The same trend was also observed for the chewing time (*p* < 0.05; Fig. 1B). The mean values for full denture were higher than those of natural teeth in two groups (*p* < 0.05; Table 1).

**Table 1:** Number of chews and chewing time according to the hardness of the test foods

Level	Hardness	Natural teeth		Full denture	
		Number of chews	Chewing time (s)	Number of chews	Chewing time (s)
Radish *Kimchi* Level 1	3.19 N	13.63 ± 5.83 ^a^	10.44 ± 3.31 ^a^	18.00 ± 10.31 ns	14.53 ± 8.09 ns^1,2^
Radish *Kimchi* Level 2	6.67 N	17.83 ± 7.13 ^ab^	13.67 ± 4.94 ^ab^	20.38 ± 12.24 ns	16.05 ± 7.00 ns
Radish *Kimchi* Level 3	9.01 N	20.17 ± 7.12 ^abc^	14.27 ± 4.57 ^ab^	25.38 ± 14.19 ns	17.06 ± 6.08 ns
Radish *Kimchi* Level 4	12.62 N	24.08 ± 8.08 ^bc^	16.65 ± 4.56 ^ab^	30.75 ± 16.72 ns	19.61 ± 7.42 ns
Radish *Kimchi* Level 5	24.48 N	27.75 ± 7.92 cd	19.40 ± 5.75 ^bc^	34.63 ± 15.70 ns	22.01 ± 7.70 ns
Radish *Kimchi* Level 6	38.66 N	34.67 ± 8.77 ^d^	24.40 ± 8.82 ^c^	42.25 ± 18.75 ns	27.05 ± 8.42 ns
F		23.88	18.48	3.00	3.02
P		< .001	< .001	0.021	0.021

One-way ANOVA results and multiple comparisons performed using the Bonferroni correction of the differences

One-way ANOVA *p* < 0.05, Bonferroni correction *p* < 0.003

^1^Values are presented as mean ± standard deviation

^2^A value in a column with different letters (a–d) differs significantly. Means in the same column with the same letter do not differ significantly

### Muscle activities according to hardness changes in radish *Kimchi*

EMG was used to measure the muscle activity among individuals, and the muscle activity across chewing duration was averaged for each test food. Throughout the mastication process, the EMG amplitude showed a significant positive correlation with radish *Kimchi* hardness. Muscle activity tended to increase with hardness (Fig. 1C and Fig. 1D), and both the masseter and temporalis muscles were significantly activated at all levels of hardness in individuals with natural teeth (*p* < 0.05).

During chewing, masticatory properties differed by group, and they significantly differed by dental status and influenced muscle activity (Table 2). Participants showed the highest muscle activity when they chewed the level-6 test food and, particularly, the masseter muscle activity differed between the older people with full dentures and those with natural teeth (Fig. 1D).

**Table 2:** Muscle activities according to the hardness changes in radish *Kimchi*

Level	Hardness	Natural teeth		Full denture	
		Channel 1,2^1^	Channel 3,4^1^	Channel 1,2	Channel 3,4
Radish *Kimchi* Level 1	3.19 N	43.88 ± 29.89 ns^2.3^	42.13 ± 17.71 ^a^	31.31 ± 11.73 ns	32.12 ± 13.20 ns
Radish *Kimchi* Level 2	6.67 N	40.80 ± 10.94 ns	45.44 ± 16.11 ^ab^	32.66 ± 12.23 ns	31.88 ± 12.59 ns
Radish *Kimchi* Level 3	9.01 N	44.47 ± 13.97 ns	46.84 ± 16.95 ^ab^	35.31 ± 13.30 ns	34.54 ± 15.64 ns
Radish *Kimchi* Level 4	12.62 N	48.04 ± 14.00 ns	52.07 ± 20.70 ^ab^	38.34 ± 11.04 ns	36.53 ± 10.16 ns
Radish *Kimchi* Level 5	24.48 N	53.99 ± 18.46 ns	56.48 ± 21.16 ^ab^	43.00 ± 11.95 ns	41.54 ± 13.10 ns
Radish *Kimchi* Level 6	38.66 N	59.92 ± 18.95 ns	68.93 ± 33.93 ^b^	47.11 ± 11.39 ns	44.89 ± 14.25 ns
F		3.53	4.72	2.11	1.27
P		0.005	0.001	0.083	0.297

One-way ANOVA results and multiple comparisons performed using the Bonferroni correctionof the differences

One-way ANOVA *p* < 0.05, Bonferroni correction *p* < 0.003

^1^Channel 1 (left temporalis), Channel 2 (right temporalis), Channel 3 (left masseter), Channel 4 (right masseter)

^2^Values are presented as mean ± standard deviation

^3^A value in a column with different letters (a–d) differs significantly. Means in the same column with the same letter do not differ significantly

As shown in Fig. 1C and Fig. 1D, the slope of the line for older people with natural teeth is steeper than that for those with full dentures. Smaller increases in muscle activity with increased hardness were observed for full-denture wearers compared to those with a natural dentition. Specifically, muscle activity was lower in older participants with full dentures than in those with natural teeth, suggesting that older people with natural teeth chewed more actively than those with full dentures. This could be due to their confidence in their proprioceptive ability.

The evaluation of masseter muscle activities, particularly for level-6 radish *Kimchi*, showed that older people with full dentures chewed less actively than those with natural teeth (*p* < 0.05).

### Comparison of tongue pressure

IOPI measurement was conducted to assess the tongue pressure of older people with different dental statuses. As shown in Table 3, the MITP was higher in participants with natural teeth (40.41 ± 16.65 kPa) than in those with full denture (22.98 ± 12.13 kPa; *p* < 0.05).

**Table 3:** Comparison of tongue pressure according to dental status

	N	Dental status	mean ± SD	*p*-value
MITP (kPa)	24	Natural teeth	40.41 ± 16.65*^ab^	0.026*
	8	Full denture	22.98 ± 12.13*	
APL (kPa)	24	Natural teeth	18.94 ± 10.27*	0.044*
	8	Full denture	10.81 ± 62.93*	

*SD* Standard deviation, *APL* Average pressure level, *MITP* Maximum isometric tongue pressure

^a^Values are presented as mean ± standard deviation

^b^Tongue pressure was measured using the Iowa Oral Performance Instrument

^*^p < 0.05, comparisons between the groups were conducted using an independent t-test

^*^p < 0.05, **p < 0.01

The mean peak values of the three records were defined as the APL. The older people with natural teeth (18.94 ± 10.27 kPa) exhibited higher tongue pressure than those with full denture (10.81 ± 62.93 kPa), and the difference was statistically significant (*p* < 0.01; Table 3). Notably, the teeth condition was positively related to the tongue muscle force. These results indicate that some foods can be compressed between the tongue and palate without being chewed by the teeth and, thus, such foods suitable for frail persons who have lost some teeth.

Subsequently, the partially texture-modified radish *Kimchi* that could be pressed between the tongue and palate was assessed.

**Fig. 2:**
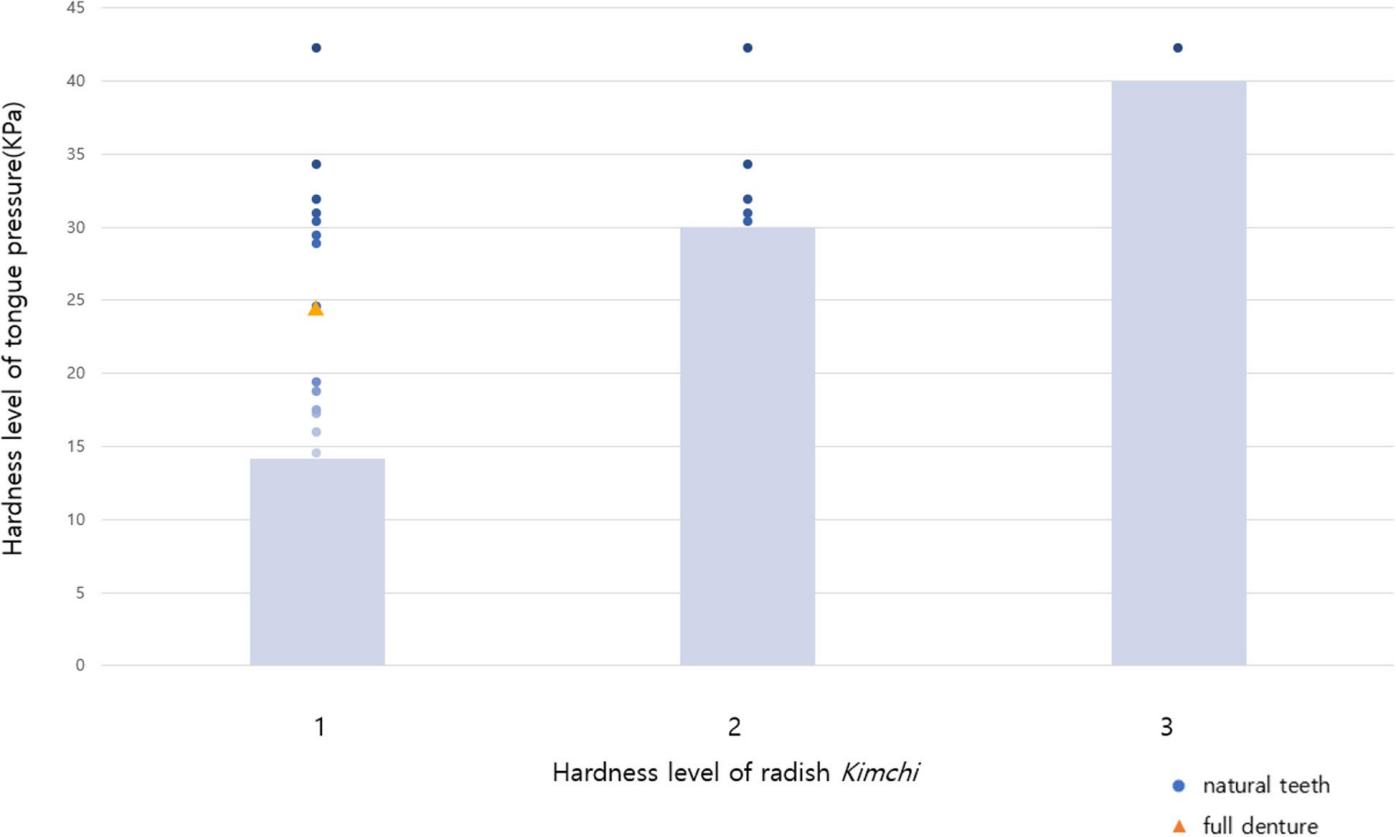
Proper tongue pressure based on the radish *Kimchi* level. The radish *Kimchi* was categorized into six levels of hardness and different textures based on texture profile analysis-measured hardness: level 1 (3.19 N), level 2 (6.67 N), and level 3 (9.01 N). Only one of the twenty four participants with natural teeth could press the level-3 radish *Kimchi*. Fourteen participants could apply the tongue pressure required to press the level-1 radish *Kimchi*, five participants could press level-2 radish *Kimchi*, and none could press the *Kimchi* with other hardness levels. In contrast, only one of the eight participants with full dentures could press level-1 radish *Kimchi*

To determine the force required to swallow, radish *Kimchi* of each level of hardness was pressed, and the unit of hardness, Newtons (N), was converted to kPa, the unit of tongue pressure. The blanched radish *Kimchi* was categorized into the following six types based on the hardness level: level 1 (3.19 N), level 2 (6.67 N), level 3 (9.01 N), level 4 (12.62 N), level 5 (24.48 N), and level 6 (38.66 N). The tongue pressure required to break down each level of radish. The blanched radish *Kimchi* was categorized into the following six types based on the hardness level: level 1 (14.2 kPa), level 2 (29.8 kPa), level 3 (40 kPa), level 4 (55.5 kPa), level 5 (108.9 kPa), and level 6 (172.9 kPa). Based on the IOPI tongue pressure measurements, only one of the twenty four participants with natural teeth could press the level-3 radish *Kimchi*. Fourteen participants could apply the tongue pressure required to press the level-1 radish *Kimchi*, five participants could press level-2 radish *Kimchi*, and none could press the *Kimchi* with other hardness levels. In contrast, only one of the eight participants with full dentures could press level-1 radish *Kimchi* (Fig. 2).

### Correlations between preference test and mastication properties

The preference test examined the participants’ acceptance of the test food based on hardness and texture. This study investigated how participants chose their favorite food among the test samples based on overall acceptability and self-reported preferences.

The sensory preferences of older participants regarding the partially blanched radish *Kimchi* were further investigated, and the correlation between the preference and masticatory performance was analyzed (Tables 4 and 5). The number of chews (0.533), chewing time (0.616) and tongue pressure (0.409) were significantly affected by preference in participants with natural teeth (Table 4). However, chewing time (0.846), temporal muscle activity (0.884) and masseter muscle activity (0.761) were significantly affected by preference in participants with full denture (Table 5) (*p* < 0.05, *p* < 0.01) (Tables 4 and 5).

**Table 4:** Pearson correlation coefficients between preference and mastication properties in natural teeth

Variables	Preference	Number of chews	Chewing time	Temporal muscle activity	Masseter muscle activity	Tongue pressure
preference	1	0.533**	0.616**	0.219	0.254	0.409*
Number of chews		1	0.383	0.347	0.110	0.256
Chewing time			1	0.352	0.219	0.315
Temporal muscle activity				1	0.506*	0.294
Masseter muscle activity					1	0.290
Tongue pressure						1

⁎ Correlation is significant with *p* < 0.05

⁎⁎ Correlation is significant with *p* < 0.01

**Table 5:** Pearson correlation coefficients between preference and mastication properties in full denture

Variables	preference	Number of chews	Chewing time	Temporal muscle activity	Masseter muscle activity	Tongue pressure
Preference	1	0.676	0.846**	0.884**	0.761*	0.278
Number of chews		1	0.893**	0.792*	0.326	0.373
Chewing time			1	0.804*	0.422	0.006
Temporal muscle activity				1	0.695	0.214
Masseter muscle activity					1	0.384
Tongue pressure						1

⁎ Correlation is significant with *p* < 0.05

⁎⁎ Correlation is significant with *p* < 0.01

### Subjective hardness scores and objective hardness levels based on the preference test

**Fig. 3:**
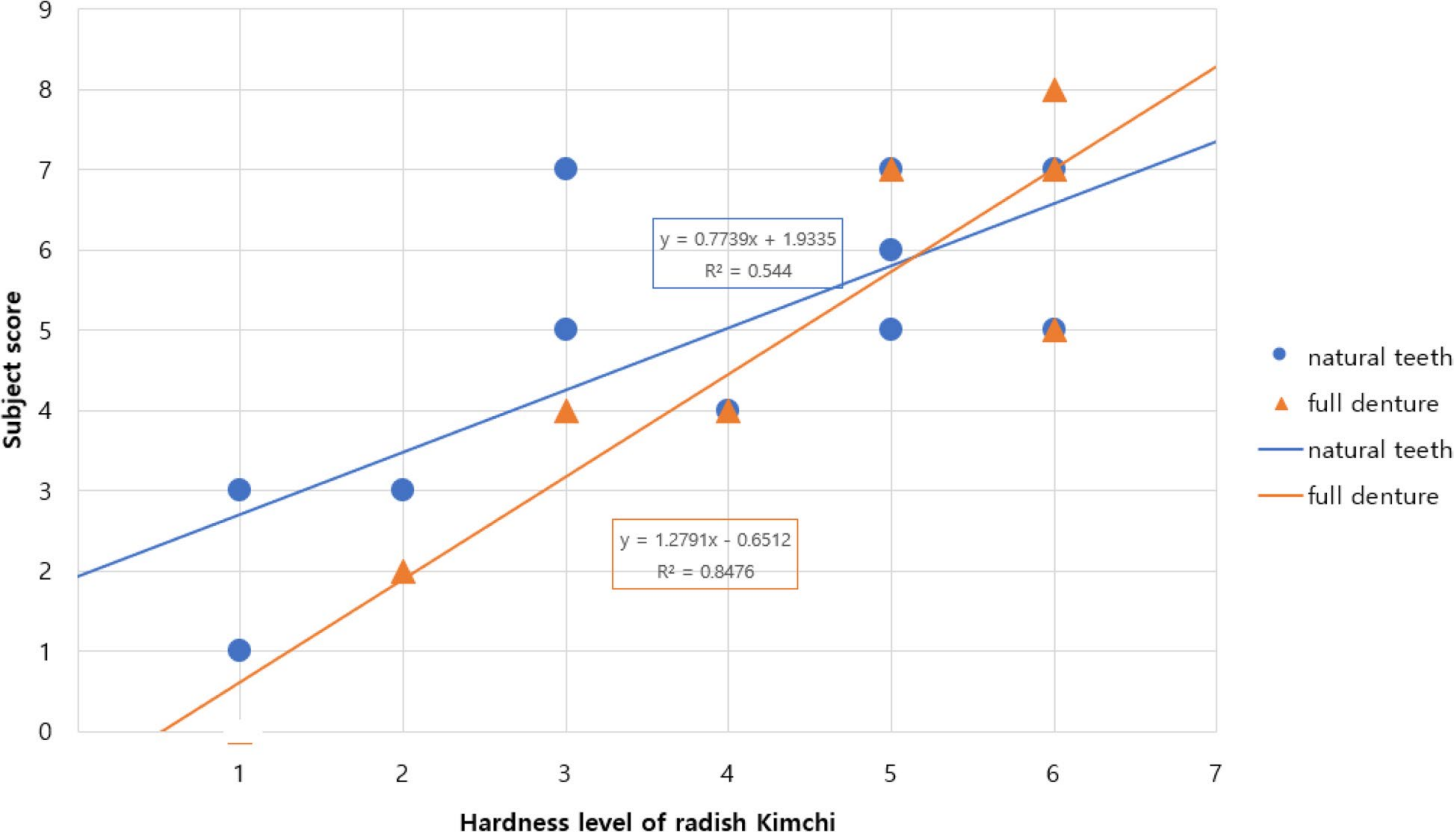
Interrelationship between the subjective score and objective level

As shown in Fig. 3, the preference pattern was different between the older people with natural teeth and those with full dentures. Indeed, all the participants tended to prefer the intact radish *Kimchi*; however, those with full dentures had difficulties in chewing hard *Kimchi* properly.

The subjective score indicates whether the sample (radish *Kimchi*) is hard or soft based on the chewing ability of the participants. An association was found between the subjective hardness score and the objective hardness level (Fig. 3). The perceived hardness intensity is linked to the chewing ability of the participant. Denture wearers exhibited a lower chewing ability, and at level 6, they perceived greater hardness of food than those with natural teeth. If a set of samples was too hard compared to their chewing ability, participants rated it with the highest score on the hardness scale.

Overall, there was no significant difference in the number of chews or chewing time between the two groups, while masseter muscle activity was significantly higher in the older people with natural teeth than in those with full dentures.

In this study, the dental status was closely related to masticatory performance. Based on these results, multiple linear regression analysis was performed to assess the relationships between masticatory performance and the dental status. The data represented the extent to which independent variables affected dependent variables. In participants with natural teeth or a full denture, the subject score was significantly related to chewing time and muscle activity (*p* < 0.05) (Table 6).

**Table 6:** Results of multiple regression analysis

Model	Unstandardized coefficient		Standardized coefficient	*t* value	*p-value*	95.0% CI for B		VIF	TOL
	B	Standard error	β			Lower limit	Upper limit		
(Constant)	3.655	1.015		3.600	0.001	1.575	5.160		
Temporal muscle activities	-0.005	0.022	-0.045	-0.240	0.813	-0.051	0.040	1.329	0.753
Masseter muscle activity	0.005	0.010	0.086	0.490	0.628	-0.015	0.025	1.134	0.882
Chewing time	0.081	0.030	0.490	2.710	0.011	0.020	0.142	1.212	0.825

Dependent variable: subject score

Independent variables: Temporal muscle activities, Masseter muscle activities, chewing time

*CI* Confidence interval

*VIF* Variance Inflation Factor, *TOL* Tolerance

*F* = 3.00, *p* = 0.047, R^2^ = 0.2434, Adj R^2^ = 0.1623, DW = 2.090

## Discussion

The aim of this study was to investigate the relationship between masticatory properties and hardness of radish *Kimchi,* which has with homogeneous texture, in older people. Participants with full dentures displayed lower masticatory muscle activity than those with natural teeth when chewing the same food, probably because of the lack of periodontal ligaments in full dentures, thereby leading to the lack of a cushioning effect [20]. Bite force measurements (a good proxy for periodontal ligament activity) are often used in dentistry because when the teeth apply force on an object, the many nerve endings innervating the periodontal ligaments can distinguish small changes in pressure [27]. Another study [27] had detected an inversely proportional relationship between bite force sensitivity and masticatory performance in a group of older participants (i.e., as the bite force sensitivity decreases, masticatory performance increases).

The effect of hardness on masticatory properties was consistent with that found in previous studies, which showed a direct and strong correlation between the number of chews, chewing time, and food hardness [8, 10, 11, 17, 20]. A previous study [8] showed that the older group chewed significantly more times than the young adult group when chewing harder samples. The dental status played an important role in distinguishing bite, oral processing time, number of chews, and liking [20].

Our findings provide substantial evidence regarding the relationship between chewing patterns and food texture, revealing that the dental status was closely related to total muscle activity. Edentulism as well as the use of nonfunctional dentures often causes chewing problems [28].

Studies have shown that masticatory dysfunction in older people is closely related to their dental status [29], and as chewing progresses, muscle activity changes depending on the hardness, adhesion, and cohesion [30]. Interestingly, the shape of the EMG signals while chewing radish *Kimchi* differed between older participants with natural teeth and those who wore full dentures. The older people with natural teeth showed a steeper curve of the EMG signal than those with full dentures; this signified quicker muscle activation. This could be due to their confidence in their proprioceptive ability. This finding supports the theory that poor fit and lack of stability are characteristics of full dentures and negatively affect masticatory function [31].

The tongue plays a role in food transport, bolus formation, and pressure control on the palate [31]. Studies have suggested an association between higher maximum tongue pressure and better masticatory ability, such as crushing [12]; accordingly, reduced tongue pressure may contribute to less efficient mastication in older people.

The relationship between tongue pressure and masticatory behavior can be explained by the role of the tongue during mastication. The tongue generates a major compressive force against the hard palate to initiate sequential swallowing actions forcing bolus through the oral-pharyngeal-esophagus tract [12]. Accordingly, tongue pressure was compared between older participants with natural teeth and those with full dentures in this study.

As described above, masticatory ability weakened with age, and participants with full dentures preferred food that could be easily broken down via tongue-palate contact, compared to the preference of those with natural teeth.

When food is soft enough for tongue-palate compression, the tongue continues pressing the ingested food until it is broken down or fractured. In contrast, if the food is too hard for tongue-palate compression, the oral strategy changes to teeth mastication for size reduction [12]. A previous study showed that the reduction in perioral muscle strength due to sarcopenia results in reduced tongue pressure [32]. Since tongue pressure is correlated with dental status, studies in this field may improve the understanding of the conditions necessary for designing hard food for older people [33].

There was a positive association between the taste preference and hardness level based on the dental status. Based on the slope of the relationship between subjective evaluations and objective instrumental measurements, hardness was different depending on the dental status. Moreover, participants with natural teeth were less sensitive to hardness than those with full dentures to the radish *Kimchi* sample, indicating a functional gap between the chewing ability and textural preference.

Mastication is influenced by individual characteristics (i.e., dental status, ethnicity, and sex) as well as food characteristics (i.e., textural qualities and food type) [34]. A previous study [15] showed that cultural backgrounds influenced food evaluations. Traditional radish *Kimchi* (*Kkakdugi*) is popular among older Koreans, who have consumed it as a prominent side dish throughout their lives. Generally, older people have well-established food preferences and are more likely to maintain traditional eating habits [15, 35]. Additionally, participants greatly preferred familiar food over unfamiliar food [36]. Other studies [7, 28] showed that oral comfort when eating was related to the sensory properties (texture) of the food.

Compared to the preference score, the subjective scores related to dietary acceptance better reflect the participants’ precise dental status. Indeed, the level 6 (not blanched, 38.66 N) radish *Kimchi* had the highest score because the participants expected the radish to be hard. Self-perceived mastication (the ability or difficulty to chew) is associated with dental status. “Preference” does not necessarily mean that a given food is the most suitable for consumption by older people [20, 35]. However, despite their preference, dental conditions often cause difficulty in chewing and swallowing solid food, such as radish [16]. Radish *Kimchi* with substantial hardness is difficult to chew by older participants with dentures. The perceived hardness intensity is linked to the chewing ability of the participant. Denture wearers exhibited a lower chewing ability, and at level 6, they perceived greater hardness of food than those with natural teeth.

Therefore, It is necessary to provide food with modified texture for older individuals with decreased eating capability. A limited blanching could be a solution to soften solid food such as radish kimchi facilitating mastication. Thus, modified-texture food should be developed to address the functional disparity between subjective scores and objective hardness levels, thereby leading to food texture acceptance. Our findings suggest that a multifaceted approach is required to satisfy both the physical and psychological needs of older consumers when developing new products for them.

This study has some limitations. First, the sample size was small, and there were limitations of sex sampling. Second, differences in occlusal forces may arise depending on the presence of natural teeth, number of teeth, and position of teeth as either anterior or posterior. Regarding the number of study participants (32 people [24 vs 8]), the total sample size was small. In a future study, we will enroll a greater number of participants. Future studies are also needed that measure the variations in occlusal forces in participants. Despite the above limitation, this research can provides a significant result of textured food masticatory performance in older people with different dental conditions.

## Conclusions

This study confirms that poor dental status is associated with decreased masticatory muscle activity, tongue pressure, and a preference for softer foods. Differences in preference among individuals reflect differences in their both physiological and psychological factors. Therefore, it is necessary to develop food products for older people that account for the state of their dentition to narrow the gap between subjective factors, such as the psychological expectation that radish *Kimchi* could be chewed with comfort and objective factors such as hardness level radish *Kimchi*. Our findings suggest that properly designed structures and sensory attributes, such as psychological satisfaction, could be key factors in developing food products for the older people. Redesigning structures without compromising the unique taste and flavor of *Kimchi* is expected to result in an ideal food product for older people.

## Data Availability

The datasets used and/or analysed during the current study available from the corresponding author on reasonable request.

## Abbreviations

EMG: Electromyography
IOPI: Iowa Oral Performance Instrument
MITP: Maximum isometric tongue pressure
APL: Average pressure level
